# Mixerless Dual-Envelope CW Radar for Displacement Sensing Using Geometric Phase Diversity

**DOI:** 10.3390/s26216908

**Published:** 2026-10-31

**Authors:** Xiaonan Jiang

**Affiliations:** Department of Electrical and Computer Engineering, University of California, Davis, Davis, CA 95616, USA; xnjiang123@gmail.com

**Keywords:** CW radar, displacement sensing, envelope detection, geometric diversity, I/Q reconstruction, mixerless radar, non-contact measurement, power detector

## Abstract

Continuous-wave (CW) radar recovers displacement from return phase, but a single envelope measurement does not provide a unique phase coordinate. This paper presents a mixerless dual-envelope (2-ENV) approach in which two spatially separated envelope detectors measure the coherent sum of the target reflection and air-coupled transmitter leakage. The leakage serves as an over-air reference, while receive geometry provides the inter-channel phase diversity needed for I/Q-like reconstruction. The reconstruction has a 1/|sin(ΔΦ)| endpoint conditioning factor, and finite-record performance also depends on operating phase and motion arc. Simulations map these effects, modulation SNR, and fully blind five-parameter fitting. A 915 MHz prototype is evaluated in sequential matched-channel and simultaneous dual-channel experiments. The matched-channel sweep verifies the predicted endpoint-conditioning trend. Simultaneous measurements show that calibration-free five-parameter fitting is record-dependent as the available motion arc changes. The calibration-aided demodulation method (CADM) estimates the channel gains and phase difference from a 20 mm amplitude record and reuses them with per-record offset refitting. Across four non-calibration records, this fixed-setup transfer yields 1.8–5.0% normalized root-mean-square error. The results demonstrate calibrated displacement recovery using an over-air reference without a mixer, wired RF reference path, or self-injection-locked oscillator.

## 1. Introduction

Non-contact displacement sensing is essential in structural health monitoring, precision manufacturing, vibration analysis, and biomedical applications such as vital-sign detection [1,2]. Continuous-wave (CW) radar offers advantages over optical and acoustic methods—it penetrates clothing, fog, and thin-walled enclosures, and operates at stand-off ranges of several meters.

For displacement sensing, the important requirement is not simply detecting motion. A single envelope or power detector can measure motion rate through the Doppler shift [3], as is well established in the radar literature [4]. Recovering the actual position waveform d(t) is harder because a single scalar channel cannot uniquely demodulate the target-induced phase and loses sensitivity near null points of the scalar sinusoidal response. In practice, this calls for phase-diverse information that can form a two-dimensional phase coordinate for displacement recovery.

**Figure 1:**
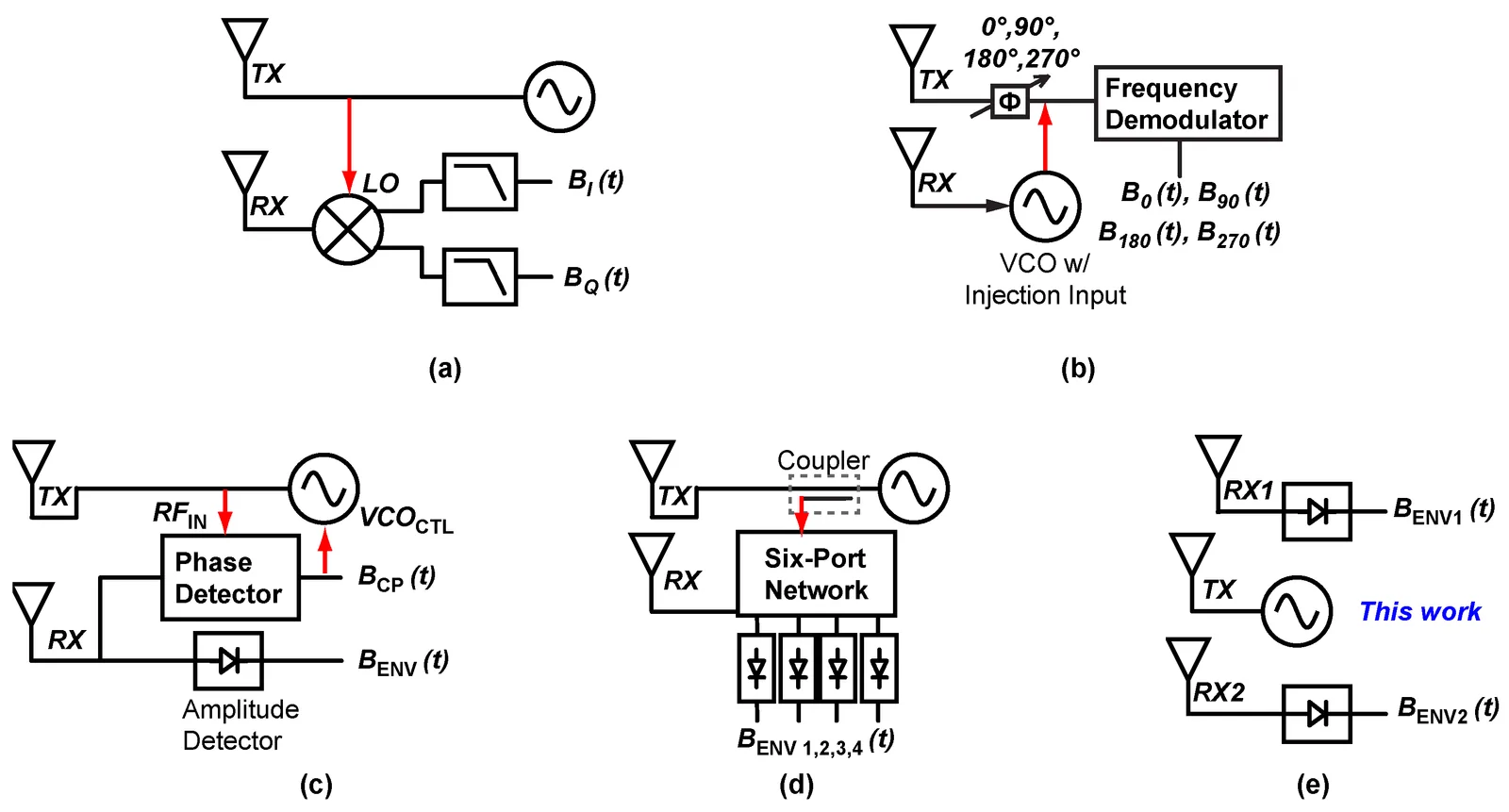
Displacement-sensing CW radar architectures. (**a**) I/Q Doppler radar: A mixer and shared LO/RF reference path (red arrow) couple the RX to the TX. (**b**) Phase-and-quadrature SIL (PQSIL): A phase shifter switches oscillator injection among four quadrature states before frequency demodulation. (**c**) Envelope-detection SIL (ENVSIL): The injected signal modulates the oscillator control path while an amplitude detector records the leakage-induced envelope component. (**d**) Six-port radar: A passive six-port interferometer combines a reference and received signal through hybrid/phase-shift paths and reads out multiple detector voltages. (**e**) This work: Two spatially separated envelope detectors observe the over-air TX leakage/coupling reference without a mixer or wired LO/RF reference path.

Representative displacement-capable CW radar architectures obtain phase diversity by coupling the transmitter (TX) and receiver (RX), as illustrated by the examples in Figure 1:

- In-phase/quadrature (I/Q) Doppler radar [Figure 1a] [5,6] achieves orthogonality through a mixer driven by a local oscillator (LO) derived from the transmitter. Low-IF heterodyne receivers instead translate the return to an intermediate frequency before digital I/Q demodulation [7]. The red arrow in Figure 1a marks this shared LO-distribution path, which constrains RX placement relative to the TX and reduces deployment flexibility.
- Self-injection-locked (SIL) radar [8,9] exploits the injection-locked oscillator as both source and detector. Related SIL displacement architectures include four-channel phase-and-quadrature SIL (PQSIL) [Figure 1b] [10] and envelope-detection SIL (ENVSIL) [Figure 1c] [3], which achieve displacement sensing through phase-switched pseudo-quadrature or amplitude–phase complementary sensing. Other SIL sensors combine a microwave differentiator and an envelope detector for vital-sign readout [11]; this is different from the complementary AM/PM readout of ENVSIL. The SIL variants considered here require the received signal to be injected back into the transmitting oscillator.
- Six-port displacement radar [Figure 1d] [12,13,14,15,16] recovers displacement via power measurements after a passive six-port interferometer forms phase-shifted combinations of the reference and received inputs. In the representative displacement-sensing implementations considered here, the received signal is combined with a reference input and read out by multiple detector ports.

Together, these architectures show that displacement recovery usually adds deliberate phase diversity through electronic quadrature, oscillator injection, or passive multiport interferometry. This paper asks whether the TX–RX leakage/coupling already present in a CW setup can instead serve as the coherent reference for an envelope-only receiver. TX–RX leakage, usually treated as an impairment in high-sensitivity CW radar [17], is useful for this purpose because it is phase-coherent with the transmitted signal. Recent ENVSIL work [3] showed that this leakage can convert motion-induced phase modulation into a measurable amplitude-modulation component. Here, the SIL oscillator path is removed; the target-plus-leakage field is instead sampled by two spatially separated envelope detectors, and receive geometry supplies the phase offset needed to construct an I/Q-like coordinate. This differs from six-port interferometry because the phase-diverse observations are produced by receive geometry rather than by a passive hybrid/phase-shift network that combines a reference input with the received signal.

In the dual-envelope (2-ENV) receiver, two spatially separated envelope detectors observe the coherent sum of target reflection and air-coupled TX leakage/coupling. The leakage/coupling term is the over-air reference; the detector locations set different static phase offsets $\Phi_{1}$ and $\Phi_{2}$. If the inter-channel phase diversity $\Delta \Phi =\Phi_{2}-\Phi_{1}$ is sufficiently large, the two envelope waveforms trace a two-dimensional manifold that can be mapped to an I/Q-equivalent coordinate for displacement recovery. Table 1 positions this architecture against representative CW displacement-sensing radar approaches. The main distinction is that the reference is radiative rather than delivered through a wired RF reference path or an injection-locked oscillator, so the geometry and reference coupling must remain stable over the measurement interval. Recent CW radar studies address self-RCS/IQ calibration in six-port receivers [18], trajectory selection for imbalance calibration [19], multi-segment offset estimation [20], and correction under attenuation modulation and multiple reflections [21]. In the proposed receiver, displacement reconstruction requires estimates of two channel offsets, two modulation amplitudes, and the geometrically determined phase separation.

The rest of the paper develops and validates this architecture. Section 2 and Section 3 derive the dual-envelope coordinate and the geometric conditions that determine whether the coordinate is observable from finite motion records. Section 4 defines the reconstruction estimators used in simulation and measurement, Section 5 maps the simulated operating space, and Section 6 validates the prototype hardware. Calibration transfer beyond the fixed setup is discussed in Section Limitations and Future Work.

The key contributions are as follows:

1. Dual-envelope displacement sensing architecture: This work introduces a mixerless CW radar that recovers displacement from two spatially separated envelope-detector outputs. The architecture removes the mixer, wired RF reference path, reference port, and SIL oscillator path, while using receive geometry to provide the needed phase diversity.
2. Geometric diversity constraints: The paper identifies three mechanisms that limit the dual-envelope coordinate: exact collapse when the inter-channel phase diversity reaches the endpoints, weak local sensitivity when a channel operates near a cosine extremum, and mirrored short-arc conditioning when the two envelope records provide nearly line-like scatter. These mechanisms show that avoiding endpoint phase-diversity collapse is necessary but not sufficient; operating phase and motion arc also determine the usable region.
3. Estimator validation: The paper evaluates three reconstruction settings for the same dual-envelope coordinate. The three-parameter blind circle fit (3P-BCF) isolates matched-channel geometry, the five-parameter blind circle fit (5P-BCF) tests fully blind unequal-channel fitting, and the calibration-aided demodulation method (CADM) tests fixed-setup recovery with calibrated channel constants. Together these evaluations separate geometric conditioning, blind fitting sensitivity, and calibrated hardware operation.

## 2. Signal Model

### 2.1. System Configuration

Consider a CW source transmitting at carrier frequency $f_{c}$ through a TX antenna illuminating a target at range $R_{0}+d\left(t\right)$, where d(t) is the displacement to be recovered. Two independent RX channels, each consisting of an antenna, a low-noise amplifier (LNA), and a linear root-mean-square (RMS) envelope detector followed by an analog-to-digital converter (ADC), observe the superposition of the target reflection and incidental TX leakage/coupling through the air. There is no wired RF reference path or shared oscillator between the TX and RX subsystems; instead, the air-coupled component acts as an effective reference without deliberate LO distribution.

### 2.2. Dual-Envelope Model

Let θ(t)=4πd(t)/λ denote the round-trip phase change caused by the target displacement. For RX channel *k*, the target reflection and the air-coupled TX leakage/coupling arrive at the envelope-detector input with amplitudes $A_{R,k}$ and $A_{\mathrm{CL},k}$, respectively. Following the amplitude-modulation relation analyzed in [3], the RF-envelope magnitude at that input is

(1) $$A_{k}^{\prime }\left(t\right)={\left[A_{\mathrm{CL},k}^{2}+A_{R,k}^{2}+2A_{R,k}A_{\mathrm{CL},k}\cos\left(\theta \left(t\right)+\Phi_{k}\right)\right]}^{1/2},\;k=1,2.$$

where $\lambda =c/f_{c}$ and $\Phi_{k}$ is the static phase of the target reflection relative to the coupling/reference component in channel *k*. Equation (1) shows that the motion-induced RF phase enters the envelope magnitude because the target reflection and coupling/reference component are coherently summed before detection.

When the target-return amplitude is small relative to the coupling/reference amplitude and the detector chain remains in its linear operating region,

(2) $$A_{k}^{\prime }\left(t\right)\approx A_{\mathrm{CL},k}+A_{R,k}\cos\left(\theta \left(t\right)+\Phi_{k}\right).$$

This first-order form is used for reconstruction; Section 5 tests it using receiver signals generated from (1). After detector conversion, baseband scaling, and DC fitting, the two digitized envelope outputs are therefore modeled as

(3) $$\begin{array}{cc} B_{\mathrm{ENV},1}\left(t\right) & =A_{1}+B_{1}\cos\left(\theta \left(t\right)+\Phi_{1}\right),\\ B_{\mathrm{ENV},2}\left(t\right) & =A_{2}+B_{2}\cos\left(\theta \left(t\right)+\Phi_{2}\right). \end{array}$$

Here, $A_{k}$ is the fitted DC level and $B_{k}$ is the fitted envelope modulation depth of channel *k*. Both are effective channel quantities, not detector-only constants. Compared with the ENVSIL case in [3], the oscillator-injection path is removed; the same amplitude-modulation relation is instead applied to two over-air receive channels.

The inter-channel phase offset is defined as

(4) $$\Delta \Phi ≜\Phi_{2}-\Phi_{1}.$$

The phase separation ΔΦ controls the diversity between the two envelope coordinates, while $\Phi_{1}$ sets the first-channel operating phase. Below, |ΔΦ| denotes the smaller separation modulo 180°, so the diversity endpoints are 0° and 180°.

This separation determines whether the second envelope channel adds independent phase information or repeats the first channel. The envelope-to-I/Q mapping below makes this dependence explicit.

### 2.3. Envelope-to-I/Q Reconstruction

Let

(5) $$x\left(t\right)≜\theta \left(t\right)+\Phi_{1},\;I\left(t\right)≜\cos x\left(t\right),\;Q\left(t\right)≜\sin x\left(t\right).$$

For the measured envelope outputs, define the normalized envelope coordinates

(6) $$u_{k}\left(t\right)≜\frac{B_{\mathrm{ENV},k}\left(t\right)-A_{k}}{B_{k}},\;k=1,2.$$

The goal of reconstruction is to obtain this I/Q-like coordinate from the two envelope outputs. Using (3) and (6), the first channel directly gives the in-phase coordinate,

(7) $$\begin{array}{cc} u_{1}\left(t\right) & =\cos x(t)=I(t), \end{array}$$

and the normalized second channel is

(8) $$u_{2}\left(t\right)=\cos\left(x(t)+\Delta \Phi \right)=I\left(t\right)\cos\Delta \Phi -Q\left(t\right)\sin\Delta \Phi .$$

Solving (8) for the quadrature coordinate gives

(9) $$\begin{array}{cc} Q(t) & =\frac{I\left(t\right)\cos\Delta \Phi -u_{2}\left(t\right)}{\sin\Delta \Phi }. \end{array}$$

Finally, after low-pass filtering the reconstructed coordinate (I(t),Q(t)), the displacement waveform is recovered up to a constant offset by standard arctangent demodulation [2]:

(10) $$\hat{d}\left(t\right)=\frac{\lambda }{4\pi }\;\mathrm{unwrap}\;\left[\mathrm{atan}2\;\left(Q_{\mathrm{LP}}\left(t\right),I_{\mathrm{LP}}\left(t\right)\right)\right].$$

Equations (7) and (9) show how the two measured envelope outputs are converted into an I/Q-like coordinate. The reconstructed I/Q samples are therefore not directly measured; they are constructed from the fitted relationship between the two envelope coordinates.

## 3. Geometric Diversity Constraint

Equation (9) separates two issues within the first-order model. If $A_{1}$, $A_{2}$, $B_{1}$, $B_{2}$, and ΔΦ are known and sin(ΔΦ)≠0, the two envelope outputs define an I/Q-like coordinate. The remaining question is when that coordinate, or the constants needed to construct it from a finite record, become poorly conditioned.

### 3.1. Coordinate Collapse

The exact dual-envelope singularity follows directly from the denominator of (9). Recovering Q(t) requires division by sin(ΔΦ), which vanishes at the two endpoint separations ΔΦ=0° and 180°. At the same endpoints, the second normalized envelope also reduces to the same or sign-reversed coordinate as the first:

(11) $$\begin{array}{cc} \Delta \Phi =0{}^{\circ} & :\;u_{1}\left(t\right)=u_{2}\left(t\right)=I\left(t\right),\\ \Delta \Phi =180{}^{\circ} & :\;u_{1}\left(t\right)=-u_{2}\left(t\right)=I\left(t\right). \end{array}$$

**Figure 2:**
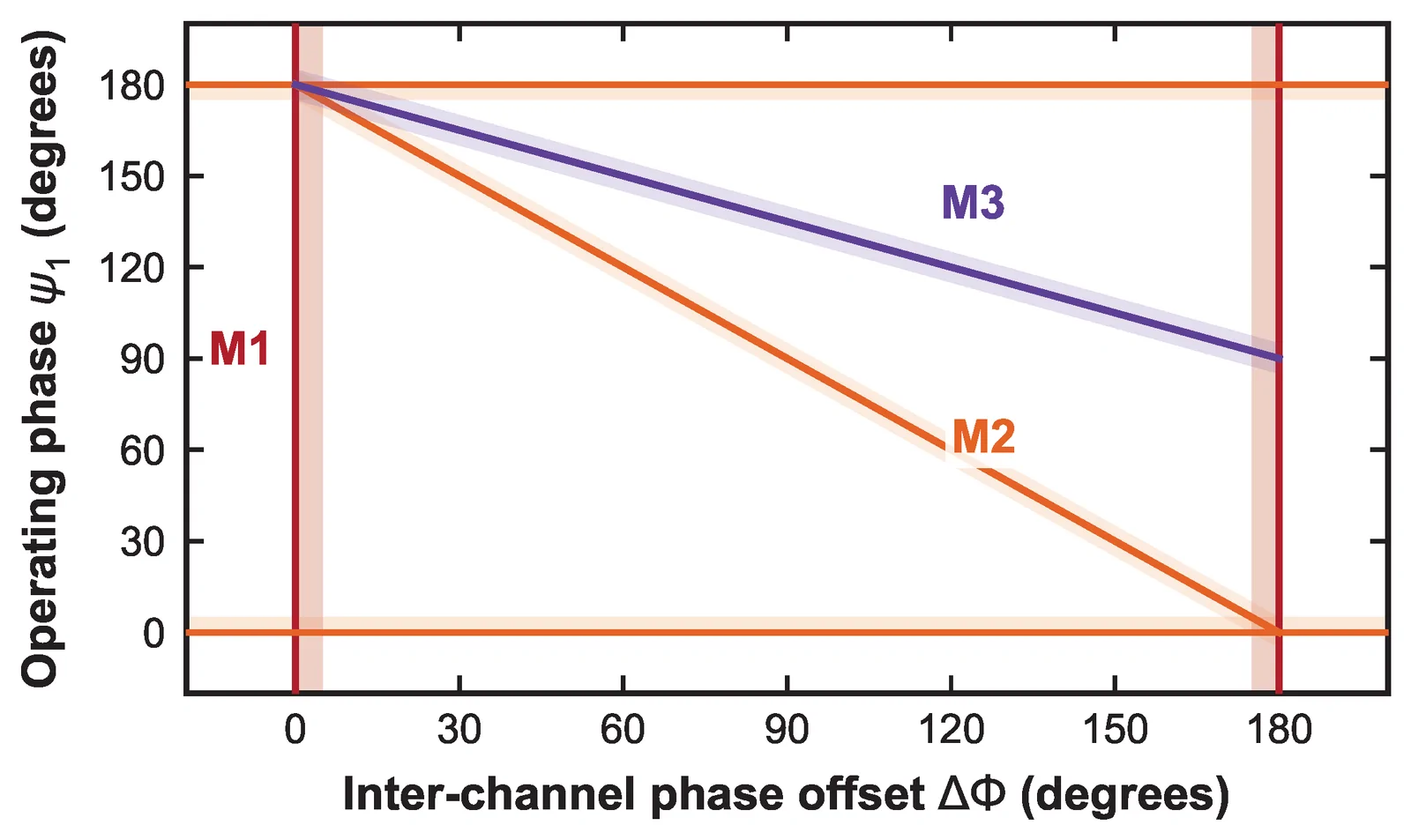
Analytical degeneracy loci in the $\left(\psi_{1},\Delta \Phi \right)$ operating space of the first-order model. M1 (red): Exact endpoint collapse, sinΔΦ=0. M2 (orange): Scalar operating-phase nulls, $\psi_{k}=0{}^{\circ}$ or 180°; in the plotted coordinates, these include the first-channel branches $\psi_{1}=0{}^{\circ}$ and 180° and the visible interior second-channel branch $\psi_{1}+\Delta \Phi =180{}^{\circ}$. M3 (purple): Mirrored short-arc conditioning; the plotted folded window shows the interior branch $\psi_{1}+\Delta \Phi /2=180{}^{\circ}$. Axes extend slightly beyond the 0°–180° folded domain to expose boundary loci.

Thus the denominator in (9) is zero exactly when the two measured waveforms no longer contain an independent quadrature coordinate. At that point, the second measured waveform is only a copy, or sign-reversed copy, of the first. The measurement pair then contains no independent sine/quadrature information: the mathematical function Q(t)=sinx(t) still exists, but it is not observable from the two envelope coordinates. Consequently, the reconstruction of Q(t) in (9) becomes indeterminate. This endpoint collapse is exact: it is set by ΔΦ rather than individual $\Phi_{k}$. It appears as the vertical M1 endpoint pair in Figure 2.

### 3.2. Operating-Phase Null Points

Away from the endpoint collapse, each scalar envelope channel inherits the legacy sinusoidal null point of CW Doppler sensing [6]: its operating phase, not ΔΦ alone, sets local sensitivity. Let $\psi_{1}$ and $\psi_{2}=\psi_{1}+\Delta \Phi$ denote the two scalar-envelope operating phases at the mean target position. For the ideal I/Q coordinate,

(12) $$\frac{dI}{dx}=-\sin x,\;\frac{dQ}{dx}=\cos x.$$

Equation (12) gives the usual I/Q complementarity [5,6]: *I* is nulled at x=0° or 180° while *Q* is sensitive, and the roles reverse at 90° or 270°. The envelope channels are not guaranteed quadrature samples; they are scalar cosines set by $\psi_{1}$ and $\psi_{2}$. For the scalar envelope coordinate in (3), the corresponding local slope is

(13) $${\left.\frac{du_{k}}{dx}\right|}_{\psi_{k}}=-\sin\psi_{k},\;k=1,2.$$

A single envelope channel is therefore weak when $\psi_{k}$ is near 0° or 180°; motion-induced RF phase then produces little envelope-amplitude change. Thus the scalar operating-phase null condition is $\psi_{k}=0{}^{\circ}$ or 180° for either channel. For channel 2, this corresponds to $\psi_{1}+\Delta \Phi =0{}^{\circ}$ or 180°. This is a scalar operating-phase null, not an exact dual-envelope singularity: with valid constants and another informative channel, (9) can still recover Q(t). In blind fitting, however, the short weak-sensitivity record poorly constrains offsets, scales, and phase separation, so finite-record conditioning depends on $\psi_{1}$ as well as ΔΦ. In Figure 2, this mechanism is M2: the first-channel branches are the horizontal loci $\psi_{1}=0{}^{\circ}$ and 180°, and the visible interior second-channel branch is $\psi_{2}=180{}^{\circ}$, equivalently $\psi_{1}+\Delta \Phi =180{}^{\circ}$.

### 3.3. Short-Arc Conditioning

Unlike the exact endpoint collapse, a finite-record ambiguity can occur when the two envelope records become local mirror images rather than a well-spread two-dimensional coordinate. Let $\psi_{c}=\left(\psi_{1}+\psi_{2}\right)/2$ be the midpoint of the two operating phases. Let $\Delta x\left(t\right)=\theta \left(t\right)-\theta_{0}$ denote the short motion-induced phase excursion about the mean range. The midpoint can take any operating-phase value. It is useful because, with $\psi_{1}=\psi_{c}-\Delta \Phi /2$ and $\psi_{2}=\psi_{c}+\Delta \Phi /2$, the two normalized envelope records are

(14) $$\begin{array}{cc} u_{1}\left(t\right)\; & =\cos\left(\psi_{c}+\Delta x\left(t\right)-\frac{\Delta \Phi }{2}\right)\\ \; & =\cos\left(\psi_{c}+\Delta x\left(t\right)\right)\cos\left(\frac{\Delta \Phi }{2}\right)+\sin\left(\psi_{c}+\Delta x\left(t\right)\right)\sin\left(\frac{\Delta \Phi }{2}\right),\\ u_{2}\left(t\right)\; & =\cos\left(\psi_{c}+\Delta x\left(t\right)+\frac{\Delta \Phi }{2}\right)\\ \; & =\cos\left(\psi_{c}+\Delta x\left(t\right)\right)\cos\left(\frac{\Delta \Phi }{2}\right)-\sin\left(\psi_{c}+\Delta x\left(t\right)\right)\sin\left(\frac{\Delta \Phi }{2}\right). \end{array}$$

Equation (14) shows that the differential sine term enters the two channels with opposite signs for any $\psi_{c}$. That sign reversal alone is not the mirror-line condition. For a short record, linearizing about Δx(t)=0 gives

(15) $$\begin{array}{cc} u_{1}\left(t\right) & \approx \cos\psi_{c}\cos\left(\frac{\Delta \Phi }{2}\right)+\sin\psi_{c}\sin\left(\frac{\Delta \Phi }{2}\right)\\ \; & \;-\Delta x\left(t\right)\left[\sin\psi_{c}\cos\left(\frac{\Delta \Phi }{2}\right)-\cos\psi_{c}\sin\left(\frac{\Delta \Phi }{2}\right)\right],\\ u_{2}\left(t\right) & \approx \cos\psi_{c}\cos\left(\frac{\Delta \Phi }{2}\right)-\sin\psi_{c}\sin\left(\frac{\Delta \Phi }{2}\right)\\ \; & \;-\Delta x\left(t\right)\left[\sin\psi_{c}\cos\left(\frac{\Delta \Phi }{2}\right)+\cos\psi_{c}\sin\left(\frac{\Delta \Phi }{2}\right)\right],\\ u_{1}\left(t\right)+u_{2}\left(t\right) & \approx 2\cos\left(\frac{\Delta \Phi }{2}\right)\left[\cos\psi_{c}-\Delta x\left(t\right)\sin\psi_{c}\right]. \end{array}$$

Thus the opposite-signed term is always present, but it becomes a mirror-line condition only when the common-mode sum is stationary to first order. From (15), this requires $\sin\psi_{c}\approx 0$, giving the periodic midpoint extrema $\psi_{c}\approx n\pi$, n∈Z. Under this condition, (15) reduces to

(16) $$\begin{array}{cc} \psi_{c} & \approx n\pi ,\;n\in Z,\\ u_{1}\left(t\right) & \approx {\left(-1\right)}^{n}\left[\cos\;\left(\frac{\Delta \Phi }{2}\right)+\sin\;\left(\frac{\Delta \Phi }{2}\right)\Delta x\left(t\right)\right],\\ u_{2}\left(t\right) & \approx {\left(-1\right)}^{n}\left[\cos\;\left(\frac{\Delta \Phi }{2}\right)-\sin\;\left(\frac{\Delta \Phi }{2}\right)\Delta x\left(t\right)\right]. \end{array}$$

The two local records therefore have equal first-order motion magnitude with opposite signs and are mirrored about a constant level. For midpoint phases away from $\psi_{c}\approx n\pi$, the common-mode term in (15) also changes with motion, so the sign flip in (14) is not sufficient to force the same mirror-line form. The traces can still show motion; the problem is that the short envelope-pair scatter approaches a line, so $A_{1}$, $A_{2}$, $B_{1}$, $B_{2}$, and ΔΦ are weakly identified from the same record. Using $\psi_{2}=\psi_{1}+\Delta \Phi$, this mirrored condition corresponds to $\psi_{1}+\Delta \Phi /2\approx n\pi$. In Figure 2, the folded operating-space window (0°–180°) shows the interior $\psi_{c}\approx 180{}^{\circ}$ branch as M3; the $\psi_{c}\approx 0{}^{\circ}$ counterpart lies on the lower folded boundary or its periodic extension. This finite-arc mirror condition is distinct from the exact vertical endpoint collapse at ΔΦ=0° and 180°.

Together, the three mechanisms in Figure 2 give a practical design interpretation rather than a single scalar rule. Moving ΔΦ away from the endpoint collapse is necessary, but the operating phase and motion arc must also be checked, especially for blind fitting. In a controlled laboratory geometry, ΔΦ can be set by the center-offset separation; in a deployed antenna configuration, it is determined by antenna geometry and should be verified from calibration data and the measured envelope scatter.

## 4. Estimator Formulations

Section 2.3 shows that dual-envelope reconstruction requires five effective coordinate quantities: $A_{1}$, $A_{2}$, $B_{1}$, $B_{2}$, and ΔΦ. This section defines three estimators for obtaining that coordinate transform from data. All three are based on the unit-circle consistency of the reconstructed I/Q samples, but they differ in which coordinate quantities are learned from each record and which are constrained or calibrated. Center estimation from short displacement arcs has also been studied in quadrature frequency-group radar [22]. Fully blind 5P-BCF is the calibration-free unequal-channel baseline: it learns all five quantities from each record. Matched-channel 3P-BCF imposes $A_{1}=A_{2}=A$ and $B_{1}=B_{2}=B$, reducing the fit to the shared offset *A*, shared scale *B*, and phase separation ΔΦ. CADM is the fixed-setup calibrated reduction: an informative calibration sweep is fit with the five-parameter model, but only ${\hat{B}}_{1}$, ${\hat{B}}_{2}$, and $\hat{\Delta \Phi }$ are retained for later records, where $A_{1}$ and $A_{2}$ are refit.

### 4.1. Five-Parameter Coordinate Model

The five quantities define the coordinate transform from measured envelopes to the reconstructed I/Q plane. The offsets $A_{1}$ and $A_{2}$ locate the two envelope-coordinate origins, $B_{1}$ and $B_{2}$ set their scales, and ΔΦ sets their relative angle. For the fixed setup studied here, $B_{1}$, $B_{2}$, and ΔΦ are treated as repeatable scale and phase-diversity constants for the target geometry and RF state. The additive origins $A_{1}$ and $A_{2}$ are allowed to change from segment to segment because detector/baseband DC baselines, record centering, and slow mean shifts can move the envelope-coordinate origin without changing the relative geometry.

In the ideal unfiltered model, enough independent samples determine a nondegenerate envelope ellipse. Its center and shape identify the five parameters, given positive scales and 0<ΔΦ<π. Short noisy arcs can still make fitting unstable; convergence of the filtered numerical fit is not guaranteed.

### 4.2. Five-Parameter Blind Circle-Fit Estimation (5P-BCF)

Fully blind 5P-BCF is the unconstrained form of the coordinate fit. It requires no separate calibration record: all five coordinate quantities are estimated from the same measurement record by minimizing the deviation of the reconstructed I/Q samples from the unit circle:

(17) $$L\left(A_{1},A_{2},B_{1},B_{2},\Delta \Phi \right)=\frac{1}{N}\sum_{n=1}^{N}{\left(I_{\mathrm{LP}}^{2}\left[n\right]+Q_{\mathrm{LP}}^{2}\left[n\right]-1\right)}^{2}.$$

The reported implementation uses data-derived bounds for offset and scale parameters, a coarse ΔΦ grid over 5°–175°, and bounded local refinement around the best grid solution.

### 4.3. Three-Parameter Blind Circle-Fit Estimation (3P-BCF)

3P-BCF is the matched-channel reference used to isolate the geometric behavior from channel imbalance. It assumes the two envelope coordinates share the same effective offset and scale, so that $A_{1}=A_{2}=A$ and $B_{1}=B_{2}=B$. The fit then has only three unknowns: *A*, *B*, and ΔΦ. Because fewer coordinate quantities are learned from the same finite motion arc, 3P-BCF is expected to be better conditioned when the matched-channel assumption is valid. It therefore represents the performance of an ideal matched-envelope implementation, while independent simultaneous receiver chains require the unequal-channel 5P-BCF model or a calibrated alternative.

### 4.4. Calibration-Aided Demodulation Method (CADM)

**Figure 3:**
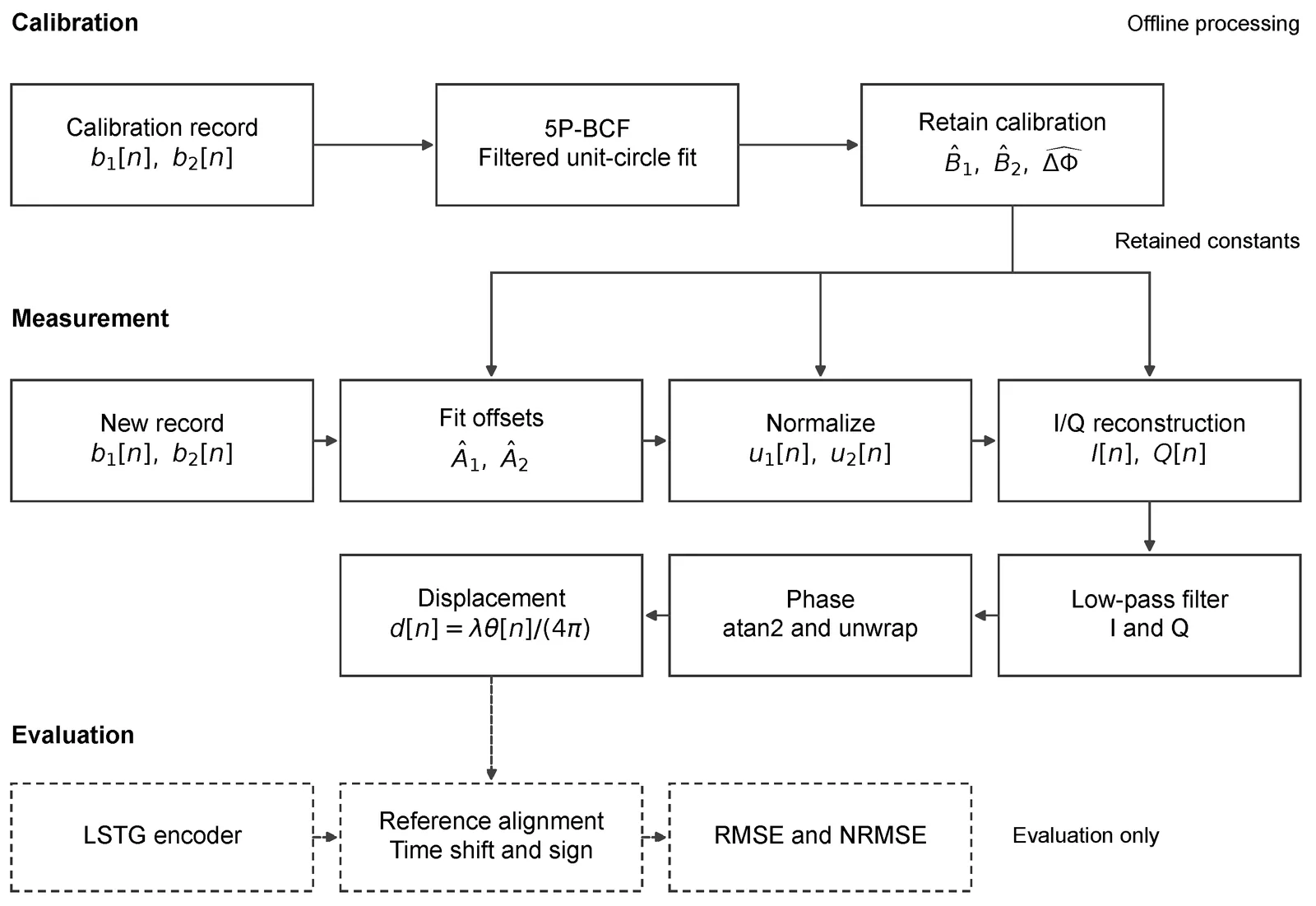
CADM calibration and displacement-recovery workflow. Calibrated $B_{1}$, $B_{2}$, and ΔΦ are retained for later records, while $A_{1}$ and $A_{2}$ are refitted. The dashed encoder-based evaluation branch is separate from reconstruction.

CADM is the fixed-setup method used when the two envelope channels are mismatched but their scale and phase-diversity constants remain stable after calibration. The processing flow is summarized in Figure 3. This reduces the short-record burden of fully blind 5P-BCF: an informative calibration sweep is used to learn $B_{1}$, $B_{2}$, and ΔΦ, and later measurement records only refit the two offsets needed to recenter the envelopes. The calibration sweep should have enough motion arc to avoid the short-arc ambiguity described in Section 3.3. The workflow is

1. Calibration sweep:
  Run 5P-BCF by minimizing $L(A_{1},A_{2},B_{1},B_{2},\Delta \Phi )$ and retain ${\hat{B}}_{1}$, ${\hat{B}}_{2}$, and $\hat{\Delta \Phi }$;
2. Measurement segment:
  Minimize $L(A_{1},A_{2}\;|\;{\hat{B}}_{1},{\hat{B}}_{2},\hat{\Delta \Phi })$ and reconstruct the resulting I/Q samples.

After offset refitting, the envelopes are normalized to reconstruct I/Q, then low-pass filtered before arctangent phase recovery, unwrapping, and conversion to displacement. The dashed evaluation branch uses the encoder only for time alignment, sign resolution, and error evaluation; it is not an input to channel fitting or reconstruction.

The reported reconstruction was performed offline in Python 3.12.7/SciPy 1.16.1 using a fourth-order 20 Hz Butterworth filter applied forward and backward for zero-phase processing. For *N* samples per channel, storage scales as O(N); the two float64 input arrays alone require 16N bytes, and sensing computation scales as O(N+EM) for *M* fitting samples and *E* total objective evaluations across solver starts and refinement, excluding encoder-based evaluation. Peak memory and end-to-end latency were not benchmarked. An embedded implementation would require bounded fitting windows and causal filtering, with accuracy and latency validated after these changes.

Section 5 next evaluates these three formulations over the operating space predicted by Figure 2 and then isolates the 5P-BCF calibration sensitivities that govern CADM transfer.

## 5. Simulation Study

**Figure 4:**
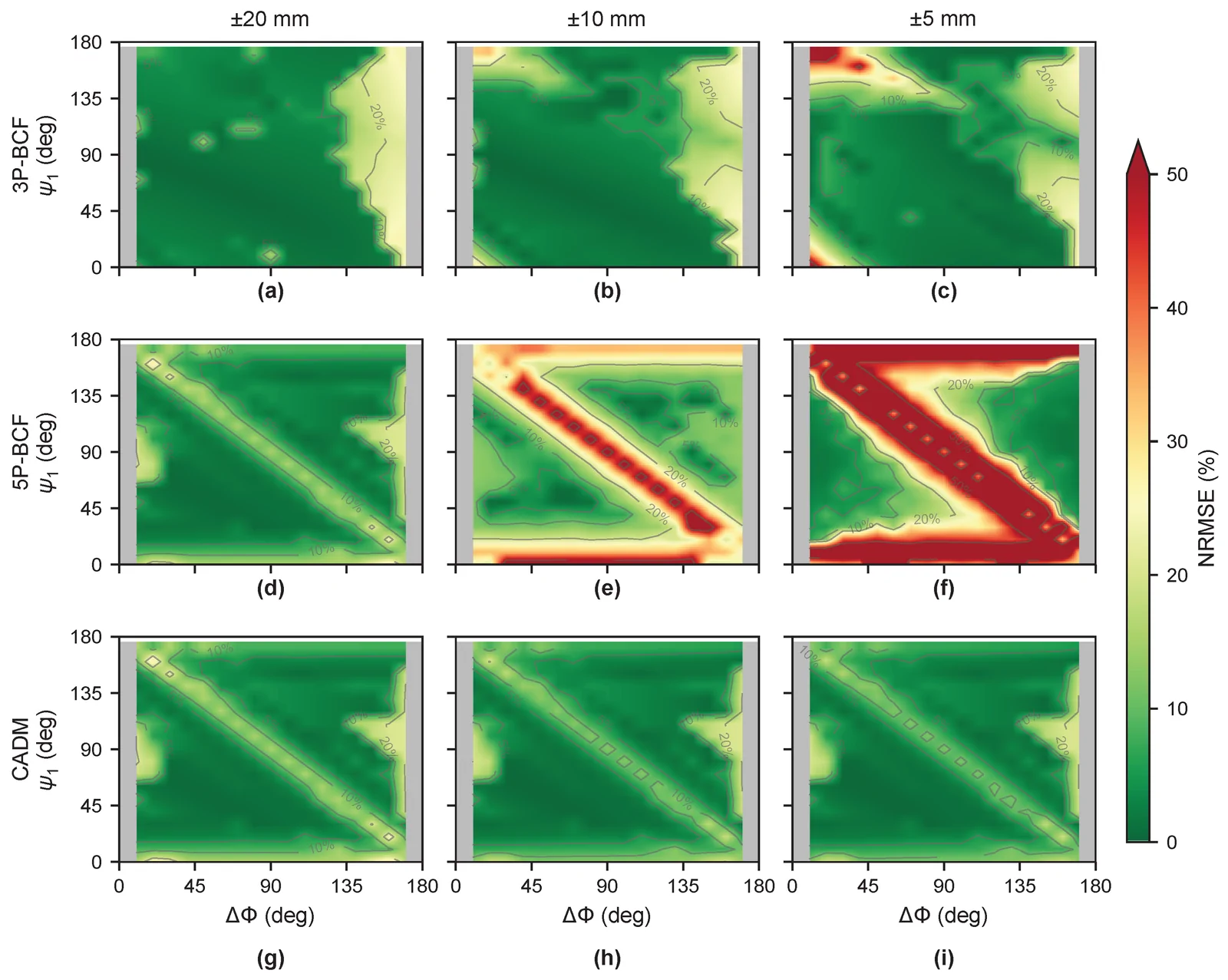
Simulation NRMSE heatmap across the sampled $\left(\psi_{1},\Delta \Phi \right)$ operating space. (**a**–**c**) Matched-channel 3P-BCF; (**d**–**f**) fully blind 5P-BCF; (**g**–**i**) CADM: For each $\left(\psi_{1},\Delta \Phi \right)$ setup, the ±20 mm record is first fit with the five-parameter model; only $B_{1}$, $B_{2}$, and ΔΦ are retained as calibration constants, and $A_{1}$ and $A_{2}$ are refit for each plotted record. Columns correspond to ±20, ±10, and ±5 mm motion amplitudes using the shared triangular record (0.5 Hz, 4 s, 1 kS/s). Gray columns mark structurally singular endpoints. Green regions indicate lower NRMSE; red regions indicate high-error configurations. Simulated at $f_{c}=915\;\mathrm{MHz}$, modulation SNR =60 dB, and assumed $A_{R,k}/A_{\mathrm{CL},k}=0.1$ in both channels.

Figure 2 first identifies the estimator-independent geometry: where the dual-envelope coordinate is singular, locally weak, or vulnerable to short-arc mirroring. Figure 4 then tests how those loci appear when the same records are processed by the three estimators used in this paper: matched-channel 3P-BCF, unconstrained 5P-BCF, and CADM. The simulation uses the operating-space coordinates and sign convention introduced in Section 3, so the heatmap can be read directly against Figure 2.

### 5.1. Setup

Unless noted otherwise, simulations use $f_{c}=915\;\mathrm{MHz}$, a deterministic 0.5 Hz, 4 s triangular displacement record sampled at 1 kS/s, with NRMSE computed as RMSE normalized by peak-to-peak displacement. For each channel, the prescribed displacement is converted to phase, θ(t)=4πd(t)/λ, and substituted into (1) to generate the received envelope. Both channels use constant RF amplitudes with an assumed ratio $\rho =A_{R,k}/A_{\mathrm{CL},k}=0.1$ (return power 20 dB below coupling). The envelopes are offset and scaled to form baseband signals, and Gaussian noise is added at the specified modulation SNR. Parameter fitting and displacement recovery follow Section 4 using the first-order model in (2). The operating-space heatmap uses one seeded noise realization per grid point, while sensitivity sweeps use 50 Monte Carlo trials per point to form the reported medians and interquartile bands. Modulation SNR is defined as $20\log_{10}\left(B_{k}/\sigma_{n}\right)$, where $B_{k}$ is the nominal first-order envelope AC amplitude. Link-budget quantities such as target-to-leakage ratio and detector range affect that amplitude, but these simulations isolate geometry, estimator burden, and measurable modulation SNR rather than sweeping link budget as an independent variable. Mapping these parameters to target distance, orientation, or antenna spacing requires a specified propagation, antenna, and target-scattering model.

**Figure 5:**
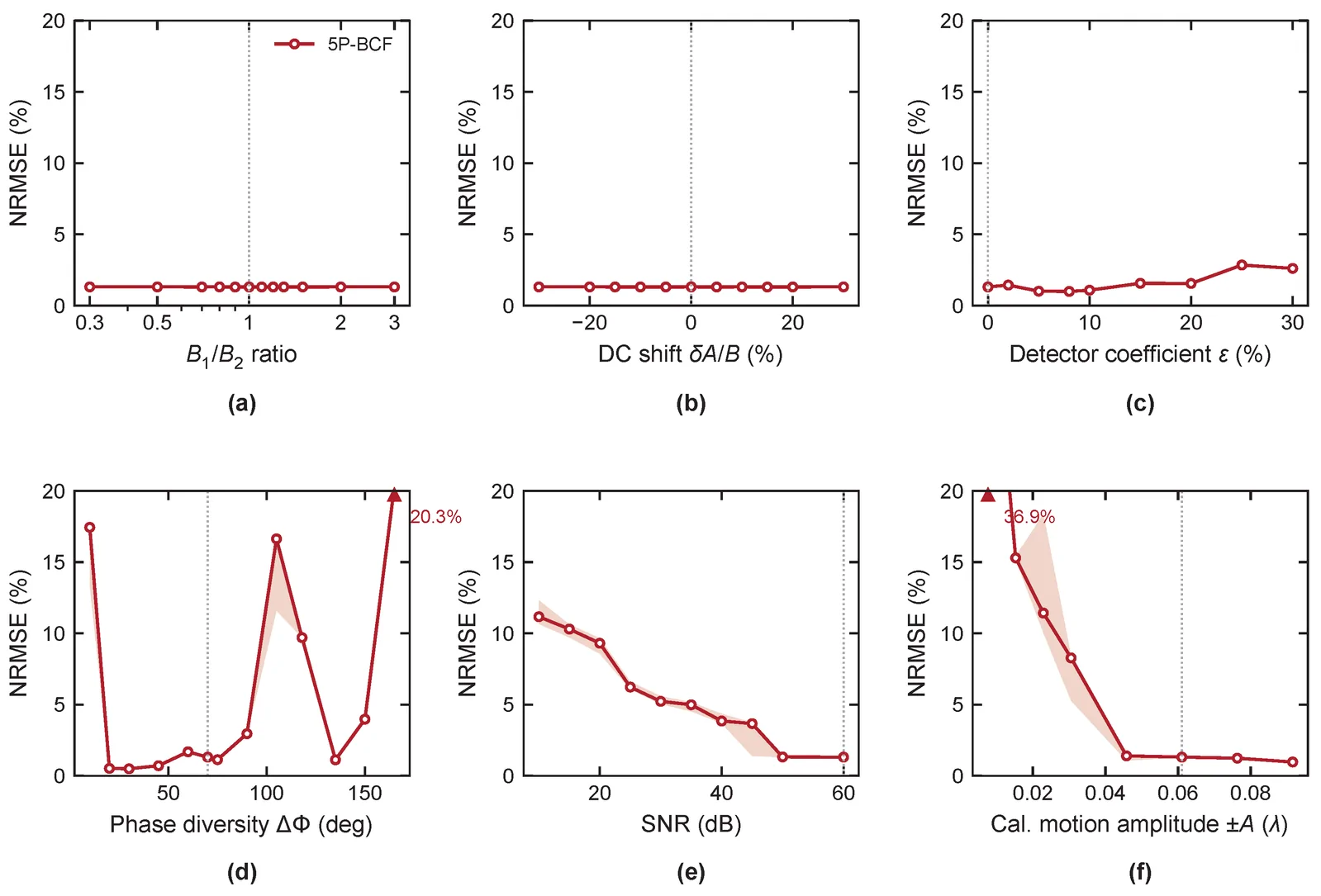
Simulated 5P-BCF calibration-sensitivity analysis at 915 MHz. Panels (**a**–**e**) use the baseline $\psi_{1}=70{}^{\circ}$, ΔΦ=70°, ±20 mm calibration motion, and 60 dB modulation SNR unless the plotted variable is swept. Panel (**f**) sweeps calibration motion amplitude over the practical short-to-moderate arc range at the same modulation SNR and record duration. Panels sweep gain imbalance, DC offset error, detector nonlinearity, phase diversity, modulation SNR, and calibration motion amplitude. Solid lines show medians; shaded regions show the 25th–75th percentile over 50 Monte Carlo trials; dotted vertical lines mark the nominal unperturbed parameter values where applicable. The assumed ratio is $A_{R,k}/A_{\mathrm{CL},k}=0.1$ in both channels. Upward triangles label medians above the 20% axis limit.

The two simulation figures have different roles. Figure 4 maps estimator behavior across the operating-space grid, while Figure 5 fixes a representative nondegenerate calibration condition and perturbs one variable at a time to test whether 5P-BCF can reliably provide the constants that CADM holds fixed during reconstruction.

For Figure 4, the grid uses ΔΦ∈{0°,10°,…,180°} and $\psi_{1}\in \left\{0{}^{\circ},10{}^{\circ},\ldots ,170{}^{\circ}\right\}$, and three motion amplitudes are tested: ±20, ±10, and ±5 mm. The second-channel phase follows the same convention as the geometric analysis, $\psi_{2}=\psi_{1}+\Delta \Phi$. The three rows in Figure 4 use this common grid to separate reconstruction assumptions without changing the motion record. The 3P-BCF row estimates *A*, *B*, and ΔΦ under $A_{1}=A_{2}$ and $B_{1}=B_{2}$. The fully blind 5P-BCF row estimates the unconstrained five-parameter coordinate from the same motion record used for displacement recovery. The CADM row uses a two-stage calibration-and-transfer procedure: at each $\left(\psi_{1},\Delta \Phi \right)$ grid point, the ±20 mm record is first fit with the five-parameter objective, and only $B_{1}$, $B_{2}$, and ΔΦ are retained. Those constants are then held fixed for the ±20, ±10, and ±5 mm records, while only $A_{1}$ and $A_{2}$ are refit for each record. Thus the ±20 mm CADM result is a calibration-record self-consistency case, whereas the ±10 and ±5 mm columns test reuse of the same constants on shorter arcs. For the detector-nonlinearity sweep, the normalized envelope $q_{k}=\left(A_{k}^{\prime }-A_{\mathrm{CL},k}\right)/A_{R,k}$ is passed through $A_{k}+B_{k}\left(q_{k}+\epsilon q_{k}^{2}\right)$ before noise is added. Channel-gain changes vary the conversion scale while retaining the assumed RF ratio.

### 5.2. Operating Space Analysis

Figure 4 is the numerical check of the three conditioning mechanisms in Section 3 under three reconstruction assumptions. Across all three rows, the endpoint-separation bands show the exact coordinate collapse predicted by (9); when sin(ΔΦ)=0, the quadrature coordinate cannot be constructed. Singular endpoints are counted as failures. The row-to-row differences show how much of the coordinate transform is learned from the same finite record. Matched-channel 3P-BCF also estimates ΔΦ, but tying the offsets and scales reduces the blind-fit burden when one channel is near the scalar operating-phase null in (13); the physical weak-slope condition itself remains. Fully blind 5P-BCF estimates independent offsets, independent scales, and ΔΦ from the same finite arc, so it is exposed to all three mechanisms; a weak-slope channel can provide little information for its own offset and scale, creating high-error pockets even inside the non-endpoint phase-diversity region. The CADM row includes the quality of the ±20 mm calibration fit, so it is not an ideal true-constant reference. Its main failures are endpoint collapse, the mirrored short-arc band described by (16), and calibration-quality structure near the same edge and diagonal loci. Calibration bias can also absorb RF-envelope model mismatch. Including the singular endpoint columns ΔΦ=0° and 180°, matched-channel 3P-BCF keeps 70%, 66%, and 63% of configurations below 10% NRMSE for the ±20, ±10, and ±5 mm records, respectively. Fully blind 5P-BCF reaches 69%, 21%, and 31%, while CADM reaches 69%, 71%, and 71% for the same three motion amplitudes. The contrast between CADM and per-record 5P-BCF on the two smaller records shows the value of transferring scale and phase-diversity constants from an informative calibration sweep rather than relearning them from each shorter arc. The ±20 mm CADM column is therefore the calibration-record case, while the two smaller-amplitude columns test reuse of those constants on shorter records. The ±5 mm count exceeding the ±10 mm count reflects operating-phase and local-minimum structure in the blind five-parameter fit rather than an improvement in fundamental conditioning. For the CADM and matched-channel rows, the low-error ΔΦ range depends on operating phase and motion arc; the minimum is not uniquely at 90°, and avoiding endpoint collapse alone does not guarantee low error. In the 5P-BCF row, additional high-error pockets remain inside this non-endpoint range because the fully blind fit is also affected by single-channel extrema and local-minimum structure in addition to endpoint collapse and mirrored short arcs.

### 5.3. Sensitivity and Operating Envelope

Where Figure 4 maps the estimator operating space, Figure 5 focuses on the upstream calibration step needed by CADM. It fixes a representative interior operating point from the low-error region of Figure 4, $\psi_{1}=70{}^{\circ}$ and ΔΦ=70°, and plots 5P-BCF alone. Once a calibration sweep has provided $B_{1}$, $B_{2}$, and ΔΦ, CADM reuses those constants on later records and refits only $A_{1}$ and $A_{2}$. The relevant question in Figure 5 is therefore whether the five-parameter calibration itself is well constrained.

Figure 5a,b shows that scale imbalance and DC offset error are not dominant for this nondegenerate, high-SNR calibration record. Because 5P-BCF fits independent offsets and scales, the median calibration error remains near 1.3% across the swept gain and offset ranges. Figure 5c shows a different effect: detector nonlinearity is a model mismatch that warps the fitted unit-circle coordinate; it interacts with RF-envelope mismatch, and the error is not monotonic in the coefficient. This sweep models a quadratic perturbation rather than a measured detector transfer curve.

Figure 5d–f shows the conditions that most strongly affect the calibration constants. The phase-diversity sweep in Figure 5d is low near the chosen ΔΦ=70° point but develops high-error pockets at other interior phase separations, consistent with the local-minimum structure seen in the 5P-BCF row of Figure 4. At 165°, median NRMSE is 20.3%; non-cosine distortion is amplified near 180° and can bias the blind fit. The SNR sweep in Figure 5e shows that the five-parameter calibration benefits directly from modulation SNR: the median error is about 3.8% at 40 dB, about 1.3% at 50 dB, and about 1.3% at 60 dB. Finally, Figure 5f isolates the calibration motion arc at fixed SNR and record duration. Very short calibration arcs poorly constrain the five unknowns, while the ±20 mm baseline lies in the low-error region for this calibration-transfer case. This motivates the CADM strategy: use an informative calibration record to estimate the geometry constants, then transfer those constants to shorter measurement records rather than relearning them with 5P-BCF each time.

## 6. Experimental Validation

The hardware validation separates the two roles established by the simulations. All measurements were conducted indoors using a single 15 cm × 15 cm flat metal plate mounted on a linear-stage actuator. The first experiment is a controlled geometry check: one receiver chain is reused while the actuator center offset is changed, so paired sequential records emulate matched envelope channels with known phase separation. This test isolates the importance of phase diversity and the endpoint conditioning trend. The second experiment uses the intended simultaneous two-channel receiver in a single fixed geometry, where the channel constants are not controlled by the center-offset sweep and must be learned from the measured data. Those records compare fully blind 5P-BCF with calibrated CADM reconstruction under unequal-channel hardware conditions. The final perturbation diagnostic then checks how sensitive CADM is to the calibrated constants used in that fixed setup. Experimental errors are evaluated against the recorded encoder waveform after mean removal, cross-correlation time alignment, and arctangent-sign resolution. Percentage error denotes NRMSE, computed as waveform RMSE normalized by peak-to-peak encoder displacement. All reported metrics use the same aligned samples and encoder excursion, without displacement-scale fitting. Calibration transfer beyond this target, range, geometry, and session is discussed in Section Limitations and Future Work.

### 6.1. Hardware

The prototype hardware and measurement components are summarized in Table 2.

In this hardware, each RX channel detects TX energy coupled through the air, which serves as the effective reference signal in the prototype. The LNA provides sufficient detector drive in this prototype; the measurements therefore evaluate the 2-ENV signal-processing geometry rather than a minimum-power detector implementation. The ADL5511 (Analog Devices, Wilmington, MA, USA) envelope-detector front end follows the hardware platform previously used for ENVSIL displacement sensing [3].

### 6.2. Sequential Matched-Channel Phase-Diversity Sweep

#### 6.2.1. Matched-Channel Test

**Figure 6:**
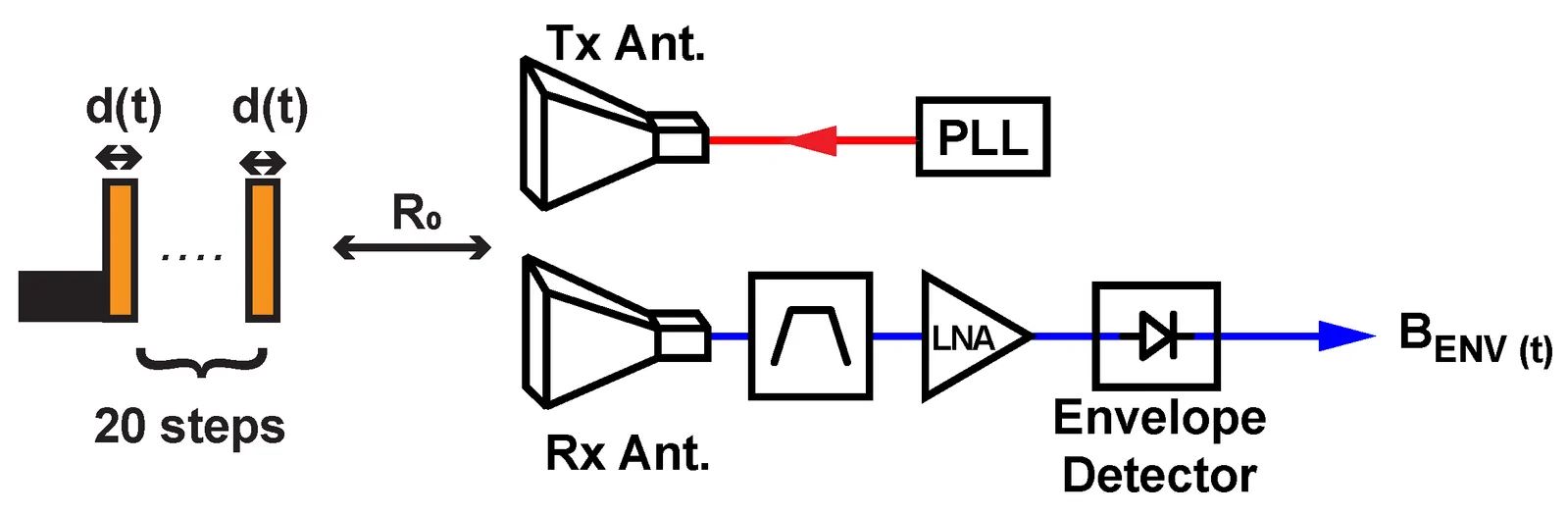
Sequential matched-channel phase-diversity sweep signal chain. A single RX channel (horn antenna, RF band-pass filter, LNA, envelope detector) is reused while the actuator center offset is changed, sequentially acquiring $B_{\mathrm{ENV}}\left(t\right)$ at 20 different operating phases to sweep ΔΦ under matched-detector conditions.

This first hardware test isolates phase-diversity conditioning from channel mismatch. The same envelope-detector receive chain is reused while the actuator center offset is stepped through 20 values from −14.5mm to +14.5mm. Pairing one anchor trace with the remaining offsets emulates matched channels ($B_{1}\approx B_{2}$) while sweeping a known ΔΦ. The signal chain is shown in Figure 6.

**Figure 7:**
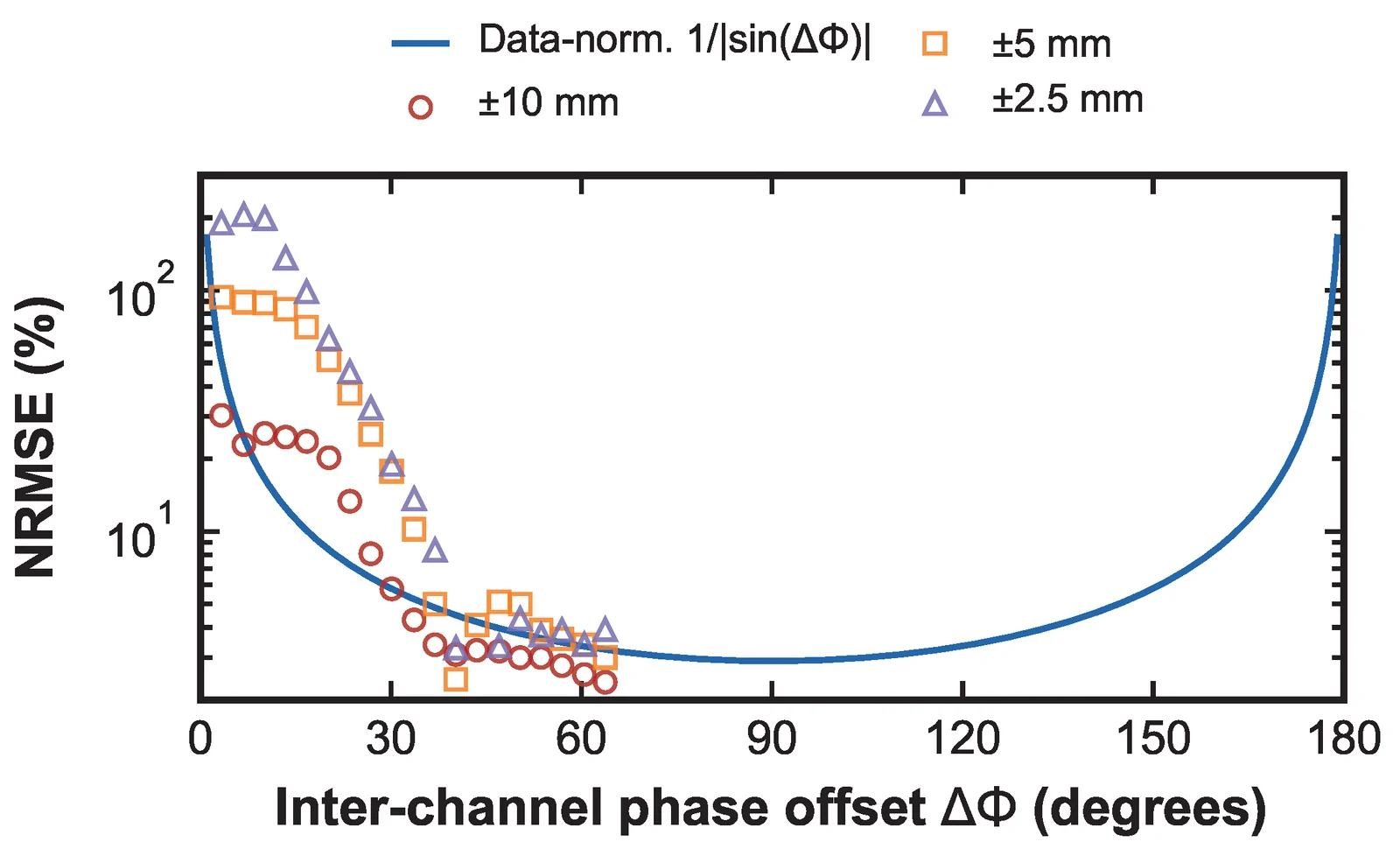
Known-geometry reconstruction NRMSE from the sequential matched-channel phase-diversity sweep versus controlled-offset phase separation ΔΦ for three actuator amplitudes in the controlled matched-channel sweep. The solid curve is a data-normalized K/|sin(ΔΦ)| endpoint-conditioning guide, with *K* set from the measured non-endpoint points to place the trend on the NRMSE scale, not to model absolute waveform error.

**Figure 8:**
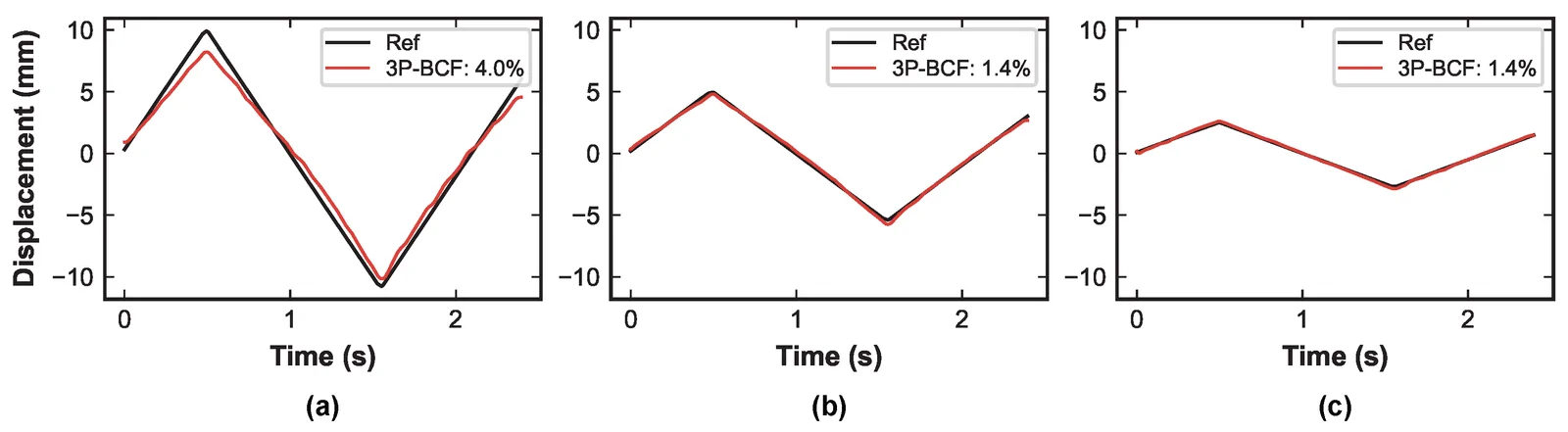
High-diversity matched-channel 3P-BCF displacement recovery from the sequential phase-diversity sweep at controlled-offset ΔΦ≈63.7°. Each panel compares 3P-BCF displacement recovery against the recorded actuator motion for (**a**) ±10 mm, (**b**) ±5 mm, and (**c**) ±2.5 mm motion amplitudes.

For each of the three motion amplitudes (±2.5, ±5, ±10 mm), one anchor trace is paired with the remaining center offsets. The same center-offset sweep is used for all three amplitudes, so the phase-separation grid is identical across the amplitude comparison. The three sessions yield 60 acquired traces and 57 nondegenerate paired configurations after excluding singular self-pairs. The actuator executes continuous-jog triangular motion, and the encoder records the actual actuator motion. Because ΔΦ is computed from the controlled center-offset separation, Figure 7 is a known-geometry check of the 1/|sin(ΔΦ)| endpoint-conditioning shape rather than a blind-estimator convergence test or absolute waveform-error prediction. The same matched-channel records are then processed with 3P-BCF for the waveform examples in Figure 8.

#### 6.2.2. Phase-Diversity Results

Figure 7 shows the measured endpoint-conditioning trend using sequentially paired matched-channel traces and the controlled-offset ΔΦ values. Self-pairs (ΔΦ=0°) are singular and are excluded from the paired NRMSE summary. The remaining points follow the expected 1/|sin(ΔΦ)| endpoint growth, while the vertical spread around the guide is expected because motion amplitude, modulation SNR, finite-record length, and processing details set the proportionality coefficient. NRMSE is computed from waveform RMSE against the recorded actuator motion, interpolated from the encoder log and time-aligned by cross-correlation with 180° arctangent-sign resolution. Thus the metric reflects recovered waveform fidelity rather than commanded-motion accuracy. Across the 19 nonsingular pairs per amplitude in Figure 7, absolute RMSE ranges from 0.490 to 6.271, 0.252 to 9.702, and 0.085 to 10.699 mm for the nominal ±10, ±5, and ±2.5 mm motions, respectively. Figure 8 provides the waveform-level counterpart to the aggregate trend in Figure 7: at a nondegenerate controlled-offset phase separation, 3P-BCF preserves the triangular displacement shape across the tested amplitudes.

### 6.3. Simultaneous Dual-Channel Experiment

#### 6.3.1. Dual-Channel Test

**Figure 9:**
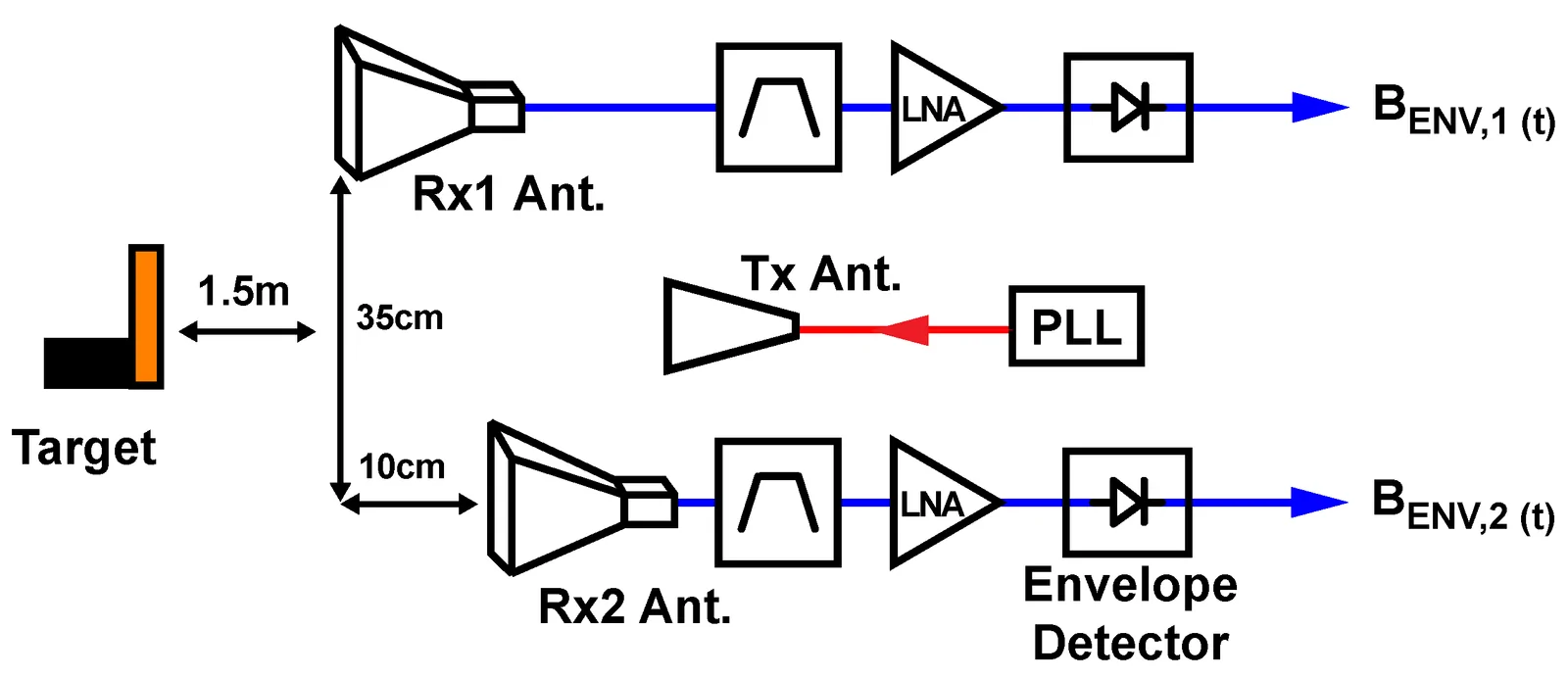
Simultaneous dual-channel 2-ENV setup. Two independent RX channels (RX1 and RX2), each consisting of a horn antenna, RF band-pass filter, LNA, and an ADL5511 envelope detector, simultaneously acquire $B_{\mathrm{ENV},1}\left(t\right)$ and $B_{\mathrm{ENV},2}\left(t\right)$. The commercial log-periodic TX antenna with 10 dB gain is positioned between the two RX antennas at 35 cm and 10 cm baselines, with the target at 1.5 m stand-off. No RF cable, shared LO, or wired RF reference path connects the TX to either RX channel.

The second hardware test uses the receiver in its intended simultaneous two-channel form. Unlike the sequential sweep, this setup does not impose a controlled ΔΦ grid; the antenna placement and over-air coupling define a single fixed geometry, and the effective scale and phase-diversity constants must be inferred from the data. Unequal channel gains make the full five-coordinate problem visible. The dual-channel setup is shown in Figure 9. The same measured records are processed two ways: 5P-BCF fits all coordinate quantities from each segment, while CADM uses a calibration sweep to fix $B_{1}$, $B_{2}$, and ΔΦ and refits only $A_{1}$ and $A_{2}$ on the remaining records.

The actuator records five motion amplitudes: ±5, ±10, ±15, ±20, and ±22 mm at 10 mm/s. The ±20 mm segment is used as the calibration record, providing a large stable sweep while the ±22 mm segment is retained as a near-limit test record. Each segment is also processed independently with 5P-BCF. For CADM, the calibration gives ${\hat{B}}_{1}=0.847\;V$, ${\hat{B}}_{2}=0.282\;V$, and $\hat{\Delta \Phi }=118{}^{\circ}$, corresponding to ${\hat{B}}_{1}/{\hat{B}}_{2}=3.0$. The fitted phase diversity is a measured outcome of this fixed setup and is away from the exact endpoint collapse. The remaining traces are reconstructed with these fixed constants and newly fitted $A_{1},A_{2}$ offsets.

#### 6.3.2. Fixed-Setup Results

**Figure 10:**
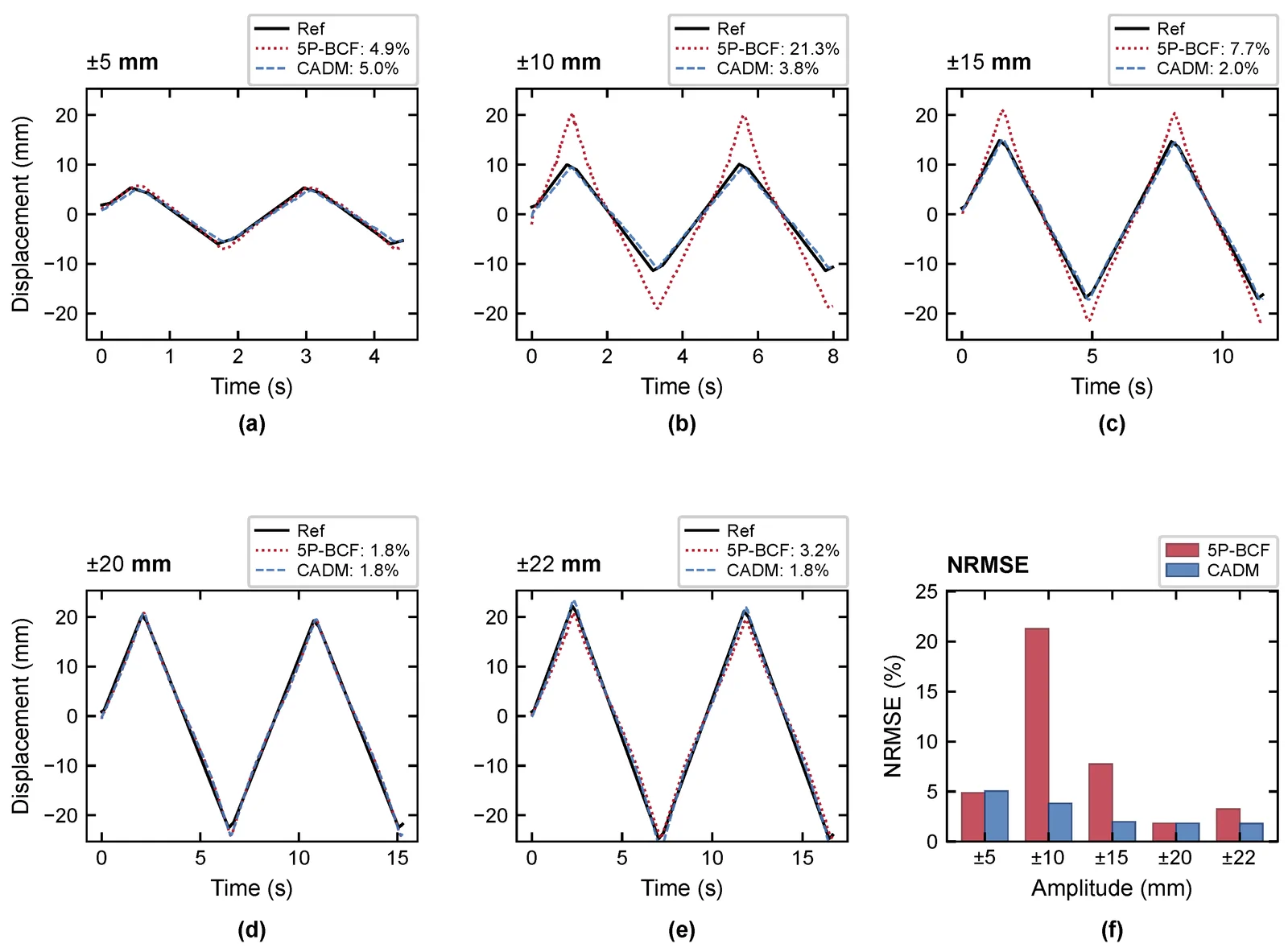
Dual-channel fixed-setup results. Panels (**a**–**e**) compare fully blind 5P-BCF and CADM displacement recovery against recorded actuator motion for five amplitudes [(**a**) ±5 mm, (**b**) ±10 mm, (**c**) ±15 mm, (**d**) ±20 mm, and (**e**) ±22 mm]. For CADM, the ±20 mm segment calibrates $B_{1}$, $B_{2}$, and ΔΦ; the other amplitudes are same-session holdouts with $A_{1}$ and $A_{2}$ refit per segment. (**f**) NRMSE summary for both methods, with the CADM calibration segment shown for reference.

The measured 5P-BCF reprocessing gives NRMSE values of 4.9%, 21.3%, 7.7%, 1.8%, and 3.2% for the ±5, ±10, ±15, ±20, and ±22 mm records, respectively. These values show calibration-free unequal-channel fitting under the measured hardware conditions. 5P-BCF is accurate on the favorable ±20 and ±22 mm records, while the larger errors at ±10 mm and ±15 mm are consistent with the short-arc, operating-point, and local-optimization sensitivities observed in simulation. This is the measured counterpart of the Figure 4 amplitude-dependence result: the hardware geometry is unchanged, but fully blind 5P-BCF must identify all coordinate quantities from each individual motion record. Using the constants from the ±20 mm calibration segment, CADM recovers the four non-calibration records with NRMSE values of 5.0%, 3.8%, 2.0%, and 1.8% for the ±5, ±10, ±15, and ±22 mm records, respectively. The corresponding CADM absolute waveform RMSE values are 0.57 mm, 0.82 mm, 0.63 mm, and 0.84 mm. The ±20 mm calibration segment gives 1.8% NRMSE and 0.78 mm absolute RMSE and is reported separately in Figure 10 as the calibration record. All plotted traces and NRMSE values are computed against the recorded actuator motion. Representative dual-channel recovery traces and the NRMSE summary are shown in Figure 10.

Table 3 summarizes RMSE, NRMSE, maximum absolute error, and Pearson correlation for the sequential and simultaneous reconstructions in Figure 8 and Figure 10, respectively. RMSE and NRMSE quantify displacement error, while correlation describes the similarity between the reconstructed and reference waveforms.

### 6.4. Measured CADM Perturbation Sensitivity

**Figure 11:**
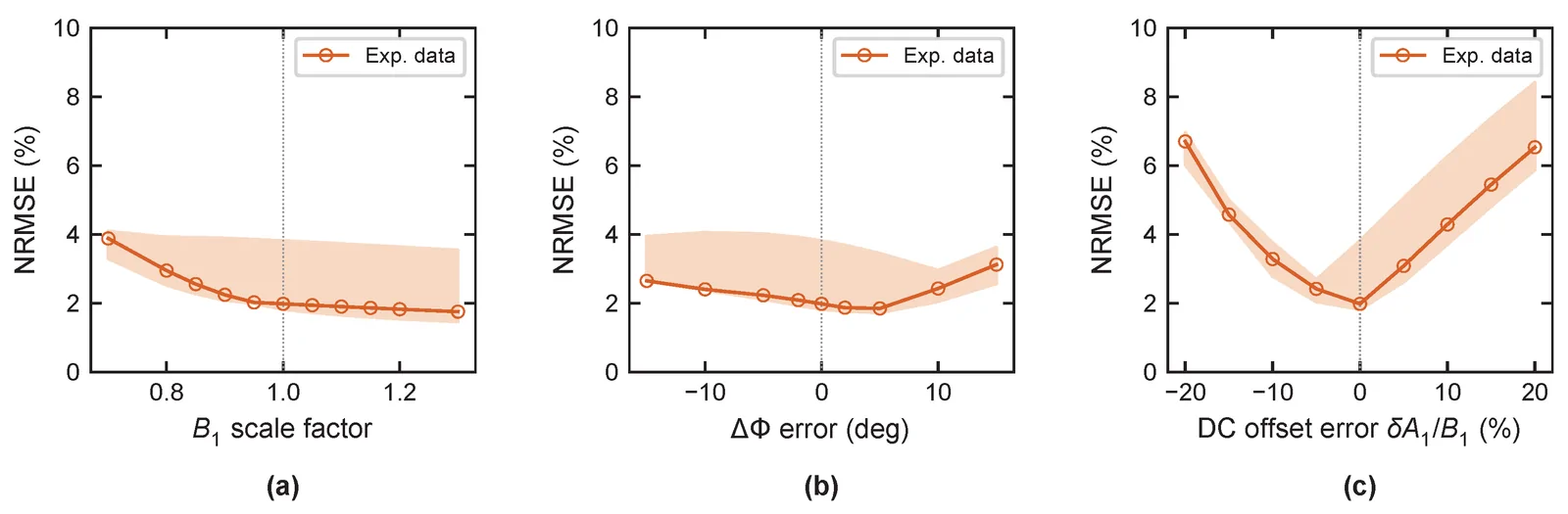
Experimental CADM calibration-sensitivity diagnostic on dual-channel hardware data. The ±20 mm segment provides calibration; shaded bands show the spread across the ±5, ±10, ±15, ±20, and ±22 mm records. Panels (**a**), (**b**), and (**c**) perturb $B_{1}$ scale, ΔΦ, and a fixed $A_{1}$ offset error, respectively. The vertical dotted lines mark the unperturbed settings: a scale factor of 1 in (**a**) and zero error in (**b**,**c**).

After the recovery comparison, the perturbation test in Figure 11 reprocesses the calibrated dual-channel records after controlled changes to selected CADM reconstruction constants. The ±20 mm segment provides the calibration, and the shaded bands summarize all five measured amplitude records (±5, ±10, ±15, ±20, and ±22 mm), including the calibration segment. This diagnostic tests sensitivity to reconstruction-constant errors on the measured data rather than serving as a holdout-recovery summary.

Scaling the calibrated $B_{1}$ by −30% increases median NRMSE from about 2.0% to 3.9%, while the tested positive scaling remains in the same few-percent error range. The phase-diversity perturbation is also mild over the tested range: even a deliberate ±15° change in ΔΦ adds at most about 1.1 percentage points to median NRMSE relative to the 2.0% baseline. These $B_{1}$-scale and phase-diversity tests indicate that moderate calibration-constant errors produce only modest degradation for this fixed target condition. By contrast, a forced $A_{1}$ offset error degrades recovery rapidly, illustrating why CADM holds $B_{1}$, $B_{2}$, and ΔΦ fixed but re-estimates $A_{1}$ and $A_{2}$ for every measurement segment. These perturbation tests also indicate sensitivity to slow environmental changes that appear as mismatches in the retained channel parameters. The calibration can be reused while the resulting displacement error remains within the application’s tolerance; determining a time interval requires the drift rate under the intended operating conditions. The two dual-channel figures serve complementary roles: Figure 10 reports fixed-setup recovery, while Figure 11 checks sensitivity to the calibrated constants used in that recovery.

## 7. Discussion

The combined results show that dual-envelope displacement recovery is governed by three coupled effects. First, receive geometry sets whether the two envelope coordinates provide enough phase diversity; near the endpoint separations, the results show the expected growth associated with the 1/|sin(ΔΦ)| conditioning in the model. Second, the matched-channel 3P-BCF results show that the same geometry can recover displacement accurately when channel scale and offset mismatch are not part of the inverse problem. Third, fully blind 5P-BCF is more demanding because the same finite motion arc must support estimation of two offsets, two scales, phase diversity, and the displacement waveform. High modulation SNR reduces noise-driven error, but it does not by itself remove this short-arc coordinate-identification ambiguity. RF-envelope mismatch can also bias the blind fit at high SNR. CADM should therefore be interpreted as a fixed-setup solution to record-dependent blind coordinate identification, not as a universal accuracy improvement. Once $B_{1}$, $B_{2}$, and ΔΦ are calibrated for the measured setup, CADM removes the most difficult blind-identification burden from each subsequent record and makes smaller-record recovery more consistent by refitting only $A_{1}$ and $A_{2}$.

The corresponding operating guidance follows directly from these effects. The receive geometry should avoid endpoint phase-diversity collapse, and the operating phase should avoid placing either envelope channel, especially both channels, at a weak local slope over the intended record. The detector input should remain in the linear range, and the motion record should provide sufficient modulation SNR and arc coverage for any blind fit. For CADM, the DC offsets should be re-estimated per segment rather than treated as fixed hardware constants. DC-offset handling is also important in DC-coupled CW displacement measurements [24]. Within a fixed target, range, geometry, and RF state, the calibrated CADM constants can be reused while displacement error remains within the required tolerance; closeness to the unit circle alone is insufficient.

### Limitations and Future Work

Frequency scaling remains a natural way to extend 2-ENV toward smaller motions. Since θ(t)=4πd(t)/λ, a higher carrier frequency gives a larger electrical phase excursion per millimeter and therefore a larger envelope arc for the same physical displacement. This can improve the finite-record conditioning that limits short motion records, provided link budget, detector linearity, and calibration stability are maintained. The practical path, however, is not simply to maximize carrier frequency; it is to choose an RF implementation that provides useful phase excursion while preserving channel selectivity and stable calibration.

The present 915 MHz implementation also reflects a practical channel-selection constraint. Because the envelope detector responds over a broad RF bandwidth, the front end must select the intended RF channel before envelope detection. The prototype uses a commercial 915 MHz RF band-pass filter ahead of the detector chain for this purpose. This filtering requirement is a practical limitation of envelope-only front ends as the concept moves beyond a single laboratory carrier. Future implementations should therefore co-design RF channel selection, detector linearity, calibration stability, and separated-device operation. Channelized communication-band transmitters, including Wi-Fi signals, are an attractive direction to evaluate because they provide practical RF channels in scenarios where a wired RF reference path or shared LO between TX and RX is undesirable.

The present validation is a controlled bench study for one target condition. The different-amplitude records test calibration reuse, not repeatability across independent acquisitions or sessions; the latter remains to be established. In that setting, the effective channel constants are calibrated from the ±20 mm record and $A_{1},A_{2}$ are refit for each segment. Stationary background returns contribute to the calibrated reference, whereas independently moving objects within the antenna beams introduce additional time-varying returns. These returns can distort the unit-circle trajectory and recovered displacement, especially when comparable to the target return, because the present prototype does not separate targets by range gating or beamforming. Adaptive beamforming in SIMO CW radar can provide spatial separation of multiple subjects [25]. Transfer across range, aspect, target reflectivity, multipath, thermal drift, or dense antenna coupling requires additional validation because those changes can alter $B_{1}$, $B_{2}$, or ΔΦ. The commanded ±22 mm segment is retained as a near-limit test record close to the usable stage stroke. Applications without a comparable known motion segment would need a separate calibration motion or adaptive self-calibration from sufficiently large natural motion, or a channelized carrier with a shorter wavelength, where the same motion spans a larger electrical arc. Follow-on prototypes should evaluate radiated multipath [26], long-duration drift, inter-channel coupling, detector linearization, and non-AWGN detector noise.

## 8. Conclusions

This paper demonstrates that two spatially separated envelope receivers can recover CW radar displacement by constructing an I/Q-like coordinate from over-air transmitter coupling and receive-path geometry. The central result is geometric: receive-path phase diversity supplies the orthogonality normally provided by quadrature hardware, while finite-record performance is set by endpoint collapse, operating phase, and short-arc conditioning, together with mismatch between the RF envelope and the reconstruction model. Equally important, the coordinate is obtained without requiring a wired RF reference path or shared LO between TX and RX, which is the deployment value for separated transmitters, multiple receivers, and future channelized communication-band implementations.

At 915 MHz, the controlled matched-channel sweep gives 57 nondegenerate paired configurations from 60 acquired traces and supports the predicted endpoint phase-diversity trend. The simultaneous dual-channel experiment, with fit-derived $B_{1}/B_{2}=3.0$, shows the record-dependence of unconstrained five-parameter fitting: measured 5P-BCF reaches 1.8% NRMSE on the ±20 mm record but rises to 21.3% on the ±10 mm record. Using the same ±20 mm segment for calibration, CADM gives consistent fixed-setup recovery on the four non-calibration records, with 1.8–5.0% NRMSE. Together with the simulation maps, these measurements separate matched-channel conditioning, fully blind unequal-channel reprocessing, and calibrated fixed-setup recovery. These results position 2-ENV as a starting point for channelized communication-band displacement sensing, where simple envelope readout and no wired/shared RF TX–RX reference path could support separated transmitters and multiple receivers. 

## Figures and Tables

**Table 1:** Comparison of displacement-sensing CW radar architectures.

Feature	I/Q	PQSIL	ENVSIL	Six-Port	2-ENV
	Radar	[10]	[3]	[13]	(This Work)
Reference/coupling route	LO path	SIL link	SIL link	Ref. port	Over-air
Wired RF TX–RX path	Yes	Yes	Yes	Yes	No
Mixer requirement	Yes	No	No	No	No
Readout channels	2	4	2	4	2
Phase diversity	Electronic	Electronic	AM/PM	Electronic	Geometry

**Table 2:** Prototype hardware and measurement components.

Component	Specification and Role
CW source	ADF4351 synthesizer (Analog Devices, Wilmington, MA, USA), 915 MHz (λ=327.64 mm).
Antennas	Commercial log-periodic TX antenna with 10 dB gain; RX horn antennas for the receive channels.
Envelope-detector chain	BPF915 915 MHz SMA band-pass filter: seller-specified 30 MHz 1 dB bandwidth, approximately 2 dB insertion loss, and rejection >58 dB at 600 MHz and >40dB at 1.2 GHz; ZX60-0916LN+ LNA (Mini-Circuits, Brooklyn, NY, USA) (20 dB, 0.7 dB NF); ADL5511 detector [23].
Data acquisition	NI USB-6001 (National Instruments, Austin, TX, USA), 14-bit, ±10 V RSE; 50 kHz (sequential sweep), 1 kHz (dual-channel CADM).
Target and motion stage	15 cm × 15 cm flat metal plate mounted on a Galil (Galil Motion Control, Rocklin, CA, USA) LSTG actuator; sequential-sweep stand-off held fixed within each record; dual-channel CADM target stand-off 1.5 m.
Stage travel	Usable LSTG stroke in the dual-channel CADM setup was approximately 45 mm; the ±22 mm record is therefore near the travel limit.
Dual-channel CADM records	±20 mm calibration sweep; ±5, ±10, ±15, and ±22 mm same-session holdouts at 10 mm/s; TX–RX baselines 35 cm/ 10 cm.
Ground truth and geometry	LSTG encoder logger, ∼145Hz; antenna baseline geometry recorded for the dual-channel CADM setup.

**Table 3:** Waveform errors for the displayed reconstructions at nominal actuator motions. The simultaneous ±20 mm record provides CADM calibration; the other simultaneous records are used for validation. *r* denotes the Pearson correlation coefficient.

Nominal Motion (mm)	Method	RMSE (mm)	NRMSE (%)	Max. Error (mm)	*r*
Sequential measurements (Figure 8)					
±10	3P-BCF	0.830	4.01	1.688	0.9979
±5	3P-BCF	0.146	1.41	0.403	0.9988
±2.5	3P-BCF	0.072	1.38	0.179	0.9997
Simultaneous measurements (Figure 10)					
±5	5P-BCF	0.551	4.86	1.480	0.9924
±5	CADM	0.572	5.05	1.238	0.9931
±10	5P-BCF	4.569	21.25	11.077	0.9930
±10	CADM	0.817	3.80	2.085	0.9973
±15	5P-BCF	2.465	7.74	6.579	0.9970
±15	CADM	0.628	1.97	1.281	0.9985
±20	5P-BCF	0.777	1.82	2.125	0.9982
±20	CADM	0.778	1.82	2.125	0.9982
±22	5P-BCF	1.519	3.24	2.616	0.9980
±22	CADM	0.843	1.80	2.807	0.9981

## Data Availability

The data and analysis code supporting the findings of this study are openly available in Zenodo at https://doi.org/10.5281/zenodo.21755773.

## Abbreviations

The following abbreviations are used in this manuscript:

2-ENV	Dual-envelope
3P-BCF	Three-parameter blind circle fit
5P-BCF	Five-parameter blind circle fit
AM	Amplitude modulation
AWGN	Additive white Gaussian noise
CADM	Calibration-aided demodulation method
CW	Continuous-wave
ENVSIL	Envelope-detection self-injection-locked
I/Q	In-phase/quadrature
LPF	Low-pass filter
NF	Noise figure
NRMSE	Normalized root-mean-square error
PM	Phase modulation
PQSIL	Phase-and-quadrature self-injection-locked
RMS	Root mean square
RMSE	Root-mean-square error
RSE	Referenced single-ended
SIL	Self-injection-locked
SNR	Signal-to-noise ratio
